# ORAL SYMPTOMS, TYPE OF MEDICAL INSURANCE, AND DENTAL CARE UTILIZATION AMONG OLDER ADULTS IN SHANGHAI

**DOI:** 10.1093/geroni/igae098.1632

**Published:** 2024-12-31

**Authors:** Xiaomin Qu, Zhihong Zhen, Xiang Qi, Bei Wu

**Affiliations:** East China University of Political Science and Low, Shanghai, Shanghai, China (People’s Republic); Shanghai University, Shanghai, Shanghai, China (People’s Republic); New York University, New York, New York, United States; New York University, New York, New York, United States

## Abstract

This study assessed the prevalence of dental care utilization among older adults in Shanghai, China. It examined the relationship between oral symptoms, the type of medical insurance, and the frequency of dental visits. Using data from the 2022 Lifelong Education for Aging Productively Survey, we analyzed responses from 1,482 participants aged 60–84. Our analysis, adjusted for complex sampling weights, reveals that only 28.93% of participants had visited a dentist in the past year. Findings from multivariable logit regression models showed that participants with oral symptoms, including toothache (OR = 2.156, 95% CI: 1.698–2.737, p < 0.01), tooth sensitivity (OR = 1.745, 95% CI: 1.375– 2.216, p < 0.01) and gum bleeding (OR = 1.275, 95% CI: 0.994–1.636, p < 0.1) were more likely to have a dental visit within the last year. The presence of multiple oral symptoms further increased this likelihood. Margins post-estimation model results demonstrated disparities in the predicted probability of visiting a dentist by the types of medical insurance. Specifically, older adults with medical insurance of less coverage were significantly less likely to seek dental care unless they exhibited multiple oral symptoms. The study concludes dental care utilization among Shanghai’s older adults is notably low and predominantly treatment-focused. Our findings underscore the urgent need for expanded dental care programs and services and broader medical insurance coverage that includes dental care to address the socioeconomic disparities in dental care access.

